# Metabolomics and gene expressions revealed the metabolic changes of lipid and amino acids and the related energetic mechanism in response to ovary development of Chinese sturgeon (*Acipenser sinensis*)

**DOI:** 10.1371/journal.pone.0235043

**Published:** 2020-06-26

**Authors:** Yanhong Zhu, Jinming Wu, Xiaoqian Leng, Hao Du, Jinping Wu, Shan He, Jiang Luo, Xufang Liang, Hong Liu, Qiwei Wei, Qingsong Tan

**Affiliations:** 1 Key Laboratory of Freshwater Animal Breeding, Ministry of Agriculture and Rural Affairs, PRC, Hubei Provincial Engineering Laboratory for Pond Aquaculture, College of Fisheries, Huazhong Agricultural University, Wuhan, China; 2 Key Laboratory of Freshwater Biodiversity Conservation and Utilization, Ministry of Agriculture and Rural Affairs, PRC, Yangtze River Fisheries Research Institute, Chinese Academy of Fisheries Science, Wuhan, China; Shanghai Ocean University, CHINA

## Abstract

Captive breeding has been explored in Chinese sturgeon (*Acipenser sinensis*) for species protection. However, gonad development from stage II to IV of cultured female broodstocks is a handicap. This study aimed to explore the physiological and metabolic changes during the ovary development from stage II to IV of female Chinese sturgeon and the related energy regulatory mechanism, which may be helpful to address the developmental obstacle. The results showed that the oocyte volume increased and the muscle lipid content decreased with the ovary development. Ovarian RNA levels of most genes related to lipid and amino acid metabolism were higher in stage II and III than in stage IV. Serum contents of differential metabolites in arginine, cysteine, methionine, purine, tyrosine, lysine, valine, leucine and isoleucine metabolism pathways peaked at stage III, while the contents of sarcosine, alanine and histidine, as well as most oxylipins derived from fatty acids peaked at stage IV. These results indicated the more active amino acids, lipid metabolism, and energy dynamics of fish body in response to the high energy input of ovary developing from stage II to III, and the importance of alanine, histidine, taurine, folate and oxylipins for fish with ovary at stage IV.

## Introduction

In female animals, reproduction and metabolism are tightly connected and reciprocally regulated. During the reproductive cycle, energy metabolism is tuned to meet the reproductive needs, which profoundly affects the mobilization of body reserves [1], including fat and protein, the major forms of energy storage in fish. Studies in human and animals have revealed that metabolites in the fasting blood used as physiological biomarkers reflect the physiological status, health and performance in vivo [2, 3]. Accordingly, the composition of plasma/serum vary with the physiological status of the animal [4]. It has been proven that fish exhibit a serial of physiological alterations as indicated by biomarkers in the blood metabolites during ovarian development [5]. Furthermore, serum metabolites are supposed to be the links between the ovarian development status of animals and the changes in body energy metabolism [6, 7].

Metabolomics has been widely used in biological studies, since it provides a directly functional readout of physiological states of an organism by differentiation, identification and quantification of small molecule metabolites [8]. In mammals, there are increasing researches on pregnancy focusing on metabolomics, including studies based on maternal serum/plasma [9] because the fasting serum components can also reflect the physiological state of the whole body. Serum metabolomics analysis in relation to the ovarian development in fish was also reported in blunt snout bream *M*. *amblycephala* [5].

Chinese sturgeon (*Acipenser sinensis*) is a typically anadromous migratory fish with a long reproductive cycle that extends for over 2 years. Wild females start spawning migration to the spawning ground in the Yangtze River when their ovaries develop to the late stage II or stage III. After then, fish stop feeding during migration, and mobilize the body reserve to meet the energy expense for migration and nutrients storage in the ovary [10, 11]. Chinese sturgeon is now a first-class protected animal in China, and a critically endangered species on the International Union for Conservation of Nature Red List due to overfishing and damming [12]. In the past 15 years, artificial breeding technologies for Chinese sturgeon have been exploring for species protection from extinction, which obtained some success [12]. However, ovary development from stage II to III or IV for the broodstocks grown up in the controlled freshwater culture system was a handicap in artificial propagation [12, 13]. Nutritional strategies, such as feeding broodstocks with fresh or frozen seawater fish [13] and optimizing the lipid level in extruded diets [14], have been shown to promote the puberty onset of Chinese sturgeon. Moreover, sufficient lipid accumulation in the ovary was supposed to be vital for successful ovarian development of Chinese sturgeon [15]. However, the physiological and metabolic changes behind the nutritional regulation on ovarian development are still unclear.

This study aimed to investigate the physiological and metabolic changes during the ovary development from stage II to stage IV in female Chinese sturgeon and the possible mechanism of ovarian nutrients deposition in relation to energy metabolism. Compared with other tissues like muscle and liver, collection of blood samples causes minimal damage to this first-class protected animal, and can meet the quantity requirement for the omics analysis. Thus, the serum was selected for metabolomics analysis in this study. The female Chinese sturgeon broodstocks (12–15 year-old hatchery produced first filial generation (F1) of wild adults with body weight of 45–61.5 kg) with ovaries at stage II, III and IV under the same nutritional condition were sampled. Biochemical components analysis and UPLC- MS based metabolic profiling in the serum, express analysis of genes related to nutrient deposition in the ovaries, as well as histological observation of muscle lipid droplet area and ovary structure were performed. The results help broaden the understanding of reproductive physiology in Chinese sturgeon.

## Materials and methods

### Experimental fish and husbandry

Chinese sturgeon broodstocks (body weight: 45–61.5 kg; body length: 175–191 cm; 12–15 year-old hatchery produced first filial generation (F1) of wild adults) were cultured in a flow-through system consisting of 6 outdoor circular pools (diameter: 12 m; water depth: 2 m) located in the Taihu Experimental Station, Yangtze River Fisheries Research Institute, Chinese Academy of Fisheries Science. There were 30 fish in each pool. Water was led into each pool at 3 L/s. Water temperature was recorded daily, and was maintained at < 26 ºC in summer and > 10 ºC in winter by mixing the different ratios of clean lake water and underground water so as to imitate the natural environment of fish during ovarian development. The pH and dissolved oxygen were monitored daily by an YSI 556 handheld multiparameter instrument (YSI Incorporated, Yellow Springs, OH, USA), and were kept at 7.0–8.0 and 6.5–7.5 mg/L, respectively. Natural light/dark regime was applied in the trial. Fish were fed with 70% artificial feed (43% crude protein, 4% fat, and 10% moisture) and 30% frozen capelins (*Mallotus villosus*, 14% crude protein, 8% fat, 75% moisture) at a feeding rate of 0.2–1.5% body weight according to water temperature.

All experimental protocols were approved by the Institutional Animal Care and Use Committee (IACUC) of Huazhong Agriculture University (Wuhan, China). Fish farming was performed according to the common Organization for Economic Cooperation and Development (OECD) protocol.

### Ultrasound, blood and tissues sampling

The females with ovaries at stage II, III and IV identified by ultrasound images were sampled following 36 h fasting during October 14–24, 2017. To alleviate the suffering of fish from sampling, sturgeons were held in a 3 m-long stretcher floating on the water with ventral side upward, water was supplied to the fish by a hose inserted into the gills to avoid hypoxia stress, and tramadol at 2 mg/kg body weight was subcutaneously injected near the surgical site before biopsy for analgesia. The ultrasonic scanning and sampling were finished in about 4 minutes. Ovarian developmental stages were identified by ultrasound detection as described by Du et al. [14]. Blood samples from 8 randomly selected females with ovary development at each stage (II, III and IV) were collected from caudal vein using a 5-ml syringe, serum were obtained by centrifugation (3000 × *g*, 10 min, 4 ºC) after clotting at 4 ºC, and stored at -80 ºC for biochemical and metabolomics analysis. Four out of 8 selected sturgeons were further subjected to the biopsy for ovary and muscle sampling according to the surgical procedures described by Du et al. [14]. Briefly, a copper probe (5 mm diameter; 30 cm length) with a long notched tube (4 mm diameter; 50 mm length) on the end was used to collect ovary and muscle tissue through a 6-mm long incision in the lateral abdominal wall. After the procedure, the incision was sealed with erythromycin ointment (XiAn Janssen Pharmaceutical Co., China). Ovary samples were split in two parts: one was fast frozen in liquid nitrogen and then stored at -80 ºC for gene expression analysis, another was fixed in Bouin’s solution for 24 h and then stored in 70% ethanol for histological analysis. Muscle samples were fast frozen in liquid nitrogen, and then stored at -80 ºC for oil-O red staining.

### Histological analysis of ovary and muscle

Ovary samples were dehydrated in titrated of ethanol solutions, cleared in a series of xylenes, and then embedded in paraffin. Sections of 5 μm thickness stained with hematoxylin-eosin (H & E) [16] were performed, observed under a ZEISS microscope (Axio Imager A2, Germany) and photographed with a CCD imaging system (Clara, Andor, UK) for further identification of sex and characterization of oocyte development. The diameters of oocytes were measured using microscopy for small oocytes and caliper for big oocytes. Muscle samples were sectioned to 8 μm on a cryostat microtome (CryoStar NX50, Thermo, USA), fixed in cold 10% formalin buffer for 10 min, stained with oil-red O for light microscopy. Three fields of each muscle sample were randomly selected for relative area (%) calculation of lipid droplets by Image-Pro Plus 6.0 [17].

### Serum biochemical analysis

The serum biochemical indices were analyzed by an automatic biochemical analyzer (Abbott Aeroset®, Abbott Laboratories, Abbott Park, IL, USA) using commercial kits (Biosino Bio-Technology Science Inc. Beijing, China). The concentrations of glucose were determined following glucose oxidase protocol (GOD) (detection range: 0.55–25 mmol/L; correlation coefficient (r) = 0.994). The concentrations of total protein were determined by biuret method (detection range: 2–100 g/L; r = 0.995). The concentrations of triglyceride were determined by GPO-PAP method (detection range: 0.3–11.29 mmol/L; r = 0.994). The concentrations of low density lipoprotein (LDL) were determined by surfactant assay (detection range: 0.30–10.00 mmol/L; r = 0.996).

### Serum metabolomics

#### Sample preparation

Serum sample (40 μl) was added to a new Eppendorf tube with ice-cold methanol (120 μl), vortex-mixed for 1 min, precipitated at -20 ºC overnight after 10 min at room temperature, and then centrifuged at 4000 × *g* for 30 min at 4 ºC to precipitate the proteins. After the centrifugation, protein free supernatant (25 μl) was transferred to a new tube and diluted with 50% methanol (225 μl) for UPLC–MS test. To assess the instrument performance, a quality control (QC) sample was prepared by thoroughly mixing 35 μl of each test sample.

#### Method development

Chromatographic separation was performed on a 2777C ultra performance liquid chromatography (UPLC) system (Waters, UK) equipped with an ACQUITY UPLC BEH C18 analytical column (100 × 2.1 mm, 1.7 μm, Waters, UK). The column oven was maintained at 50 ºC. The mobile phase consisted of solvent A (water + 0.1% formic acid) and solvent B (acetonitrile + 0.1% formic acid). Gradient elution was set as follows: 0–2 min, 100% phase A; 2–11 min, 0% to 100% B; 11–13 min, 100% B; 13–15 min, 0% to 100% A at a flow rate of 0.4 ml/min. The injection volume for each sample was 10 μl. The metabolites eluted from the column were detected by a high-resolution tandem mass spectrometry Xevo G2 XS QTOF (Waters, UK) with an electrospray ionization source (ESI) operating in positive and negative ion mode. The capillary voltages were set at 3 kV (+) and 2 kV (-), respectively, and the sampling cone voltage was set at 40 V for both modes. The mass spectrometry data were acquired in Centroid MSE mode. The TOF mass range was from 50 to 1200 Da and the scan time was 0.2 s. During the acquisition, the leucine-enkephalin (LE) signal was acquired every 3 s to calibrate the mass accuracy. Furthermore, QC samples were periodically analyzed throughout an analytical run in order to provide robust quality assurance for each metabolic feature detected. Ten QC samples before the first test sample, a QC sample after every 10 test samples, and 3 QC samples at the end were run to calibrate for the drift in the retention time of all analyses due to the matrix effect.

#### Data processing and validation

Raw data obtained from UPLC-MS were converted to NetCDF format using Masslynx version 4.1 (Waters Corp., Manchester, UK), and then were imported into Progenesis QI software (version 2.2) to construct a matrix of mass-to-charge ratio (m/z) versus retention time (RT) versus ion intensity for chemometric analyses. Relative standard deviation (RSD) was calculated for all metabolic features in QC samples, and the low-weight ions with RSD > 30% were removed from the extracted data to ensure the metabolic quality. In addition, the QC-RLSC (Quality control-based robust LOESS signal correction) method was employed to correct the data. In order to adjust the importance of high and low abundance metabolites to an equal level, the data was preprocessed by conversion of log2 and Pareto scaling prior to univariate and multivariate analyses [18].

Univariate analysis was performed by *t*-test and fold change analysis using R language analysis package [19]. In the process of statistical analysis, the p-value was acquired from the *t*-test and was corrected using the false discovery rate (FDR) method to get the q-value. Multivariate analysis was performed by principal component analysis (PCA) to validate the quality of analytical system and to observe possible outliers. Meanwhile, partial least squares-discriminate analysis (PLS-DA) was applied to establish a relational model between experimental samples and the higher abundance amount to create a model prediction for different samples, and the results were visualized in score plots to display the group clusters. The Q^2^ (predicted variation) and R^2^ (explained variation) parameters were used to evaluate the models. In addition, the corresponding influence intensity and explanation capacity of each metabolite’s higher abundance mode effects for sample groups was estimated by the value of variable importance of projection (VIP). Following standard protocol, the robustness of the final PLS-DA model was further validated by comparing the R^2^ value to a reference distribution of all possible models using permutation testing (n = 200) [20]. Metabolites in serum among groups were differentiated by the following criteria: a VIP value more than 1, a q-value less than 0.05, and a fold change value more than 1.2 or less than 0.8. Metabolites and its pathways were identified using the Kyoto Encyclopedia of Genes and Genomes (KEGG, http://www.kegg.jp/).

### Quantitative real-time PCR

Gene expressions in the ovarian samples (n = 4) were detected by quantitative real-time PCR (qPCR), including 16 genes related to lipid metabolism, 8 genes involved in yolk protein and amino acids metabolism. Total RNA was extracted, quantified spectrophotometrically, and the integrity was assessed by agarose gel electrophoresis. The cDNA was synthesized using a PrimeScript^TM^ RT reagent Kit with gDNA Eraser (TakaRa, Dalian, China). The suitability of internal standard genes was assessed by agarose gel electrophoresis of relative quantity PCR product. The qRT-PCR was carried out by a quantitative thermal cycler (MyiQTM 2, BIO-RAD, CA, USA) in 20 μl reaction volume using SYBR® Green Realtime PCR Master Mix (TOYOBO, Osaka, Japan) according to the manufacturer’s instruction. Primers in Table 1 were designed through Primer Premier 5.0 software according to gene sequences obtained from NCBI. The qRT-PCR parameters were composed of initial denaturation at 95 ºC for 30 s, followed by 40 cycles at 95 ºC for 5 s, 57 ºC for 10 s and 72 ºC for 15 s. The expression level of each tested gene was normalized to *β-actin* and *18s-RNA*, and calculated by the 2^-∆∆CT^ method [21].

**Table 1:** Quantitative polymerase chain reaction (q PCR) primer sequences used for gene expression assays.

Gene name	Accession No.	Description	Sequences of primers (5’-3’)
*β-actin*	AJ745100.1	Forward	GGTGTCATGGTTGGTATGG
		Reverse	GCTCATTGTAGAAGGTGTGA
*18s-RNA*	AY904452.1	Forward	CAGATACCGTCGTAGTTCC
		Reverse	CCTTCCGTCAATTCCTTTAAG
*camkk2*	MK474620	Forward	GGAGGTGGAGAACTCTGTCAA
		Reverse	TTGCTTCCTGTGGCTACTGT
*tab1*	MK474621	Forward	CGATGATTGGCTTAGACTTGGA
		Reverse	CCGCTGAGAACGAGGATGA
*pp2ac*	MK474622	Forward	TCACCAGACACCAACTACCTT
		Reverse	TCCATACTTCCTCAGACACTCA
*acc2*	MK474623	Forward	CAGCGTACAGGCAGGTCTT
		Reverse	ACAAGGTGGTGGTGGAGTG
*gpat3*	MK474624	Forward	GCCAACCATAGCATAGCA
		Reverse	GCGAACTTGTCCATCTGA
*dgat2*	MK474625	Forward	GATGACCTCCTTCCTCCTTGT
		Reverse	AGCGTATTCTGGCGTGTTAC
*srebp1*	MK474626	Forward	ATCGCCAAGCCAGAGAATGAG
		Reverse	TCACTGCCACCACCACCAT
*pparγ*	MK474627	Forward	CTACGGAGCGGAACTGACA
		Reverse	CCAGCGATATTGACCAGATGAA
*pparα*	MK474628	Forward	AATTTGCTTGGCGAGGGTTT
		Reverse	GGTTCTTCAGGAGGACAATACG
*lpl*	FJ436088.1	Forward	CTCAGCAGCAGCAGCATTAC
		Reverse	GTGTCATCGTCAGGAAGTTCAG
*fatp1*	MK474629	Forward	TAACCACTGGACCACACT
		Reverse	GACCTATACTTGCGGACATA
*fabp3*	MK474630	Forward	CCAGAGCACCTTCAAGAACAC
		Reverse	GCACCAGCGATGTCTCCTT
*cpt1a*	MK474631	Forward	ACACAATCCACGCCATCCT
		Reverse	TACATCCACACCTTGAAGAAGC
*mcad*	MK474632	Forward	GCAATGTCACCAGCGAAT
		Reverse	AACACCAGGCAGTCTCAT
*ucp2*	MK474633	Forward	CCGTCAAGCAGTTCTACA
		Reverse	ATGGCATTCCGAGCAATA
*pgc1α*	MK474634	Forward	GAGGAATACCAGCACGAGAGG
		Reverse	GCAGCGAAGGCATCACAAG
*vtgr*	MF631001	Forward	CTGTGTTGCTATTGGCTATG
		Reverse	CTGGATGTTGCTCTGGTAG
*vha*	MH745132	Forward	GTATCTGGCTGTTGGCATTCA
		Reverse	GCATATTCTTGGCGTGTCTCTT
*ctsd*	MH580286	Forward	GCGTTCATTGCTGCCAAGT
		Reverse	GCGTGTGATGTTCAGGTAGTT
*cat3*	MK474635	Forward	TTCGGACCATCACTGACA
		Reverse	GACAGACAGACAGACAGAC
*gatm*	MK474636	Forward	CGGTATGCTCGGTATTCAA
		Reverse	CAGAAGTATGGCGGTCAAT
*crt1*	MK474637	Forward	GGCGGTCATCATCTGGAATAC
		Reverse	TCACAGTAAGAGCAGGAATGGA
*arginase2*	MK474638	Forward	CCGACGACGACACCTACAA
		Reverse	TGAGCAGGAAGGACACAGATT
*odc1*	MK474639	Forward	AACCGCACAGAACATAACT
		Reverse	GACATAGCAGAACCACCAA

*camkk2*: calcium/calmodulin-dependent protein kinase kinase 2; *tab1*: TGF-beta activated kinase 1 binding protein 1; *pp2ac*: serine/threonine-protein phosphatase 2A catalytic subunit; *acc2*: acetyl-coA carboxylase 2; *gpat3*: mitochondrial glycerol-3-phosphate acyltransferase 3; *dgat2*: diacylglycerol O-acyltransferase 2; *srebp1*: sterol regulatory element-binding protein 1; *pparγ* and *pparα*: peroxisome proliferator-activated receptor γ and α; *lpl*: lipoprotein lipase; *fatp1*: fatty acid transport protein 1; *fabp3*: fatty acid binding protein 3; *cpt1a*: carnitine O-palmitoyltransferase 1A; *mcad*: medium-chain specific acyl-CoA dehydrogenase; *ucp2*: mitochondrial uncoupling protein 2; *pgc1α*: peroxisome proliferator-activated receptor γ coactivator 1α; *vtgr*: vitellogenin receptor; *vha*: vacuolar-type H^+^-ATPase; *ctsd*: cathepsin d; *cat3*: cationic amino acid transporter 3; *gatm*: glycine amidinotransferase (L-arginine:glycine amidinotransferase); *crt1*: sodium- and chloride-dependent creatine transporter 1; *odc1*: ornithine decarboxylase 1.

### Statistics

The data obtained from histological analysis, serum biochemical analysis, quantitative real-time PCR were analyzed using one-way ANOVA to detect the significance among three development stages, and means were compared using Tukey’s multiple range tests with SPSS (Version 19.0, SPSS Inc). The p-values acquired from the multiple range tests of qRT-PCR data were corrected using the FDR method to get the q-values. Prior to statistical analysis, the normality of all data was approved by the Kolmogorov-Smirnov test. Values were expressed as mean ± SEM and the probability value of *P* < 0.05 (q < 0.05 in qRT-PCR data analysis) was considered as significant.

## Results

### Morphological and histological changes in the ovary during development

**Fig 1:**
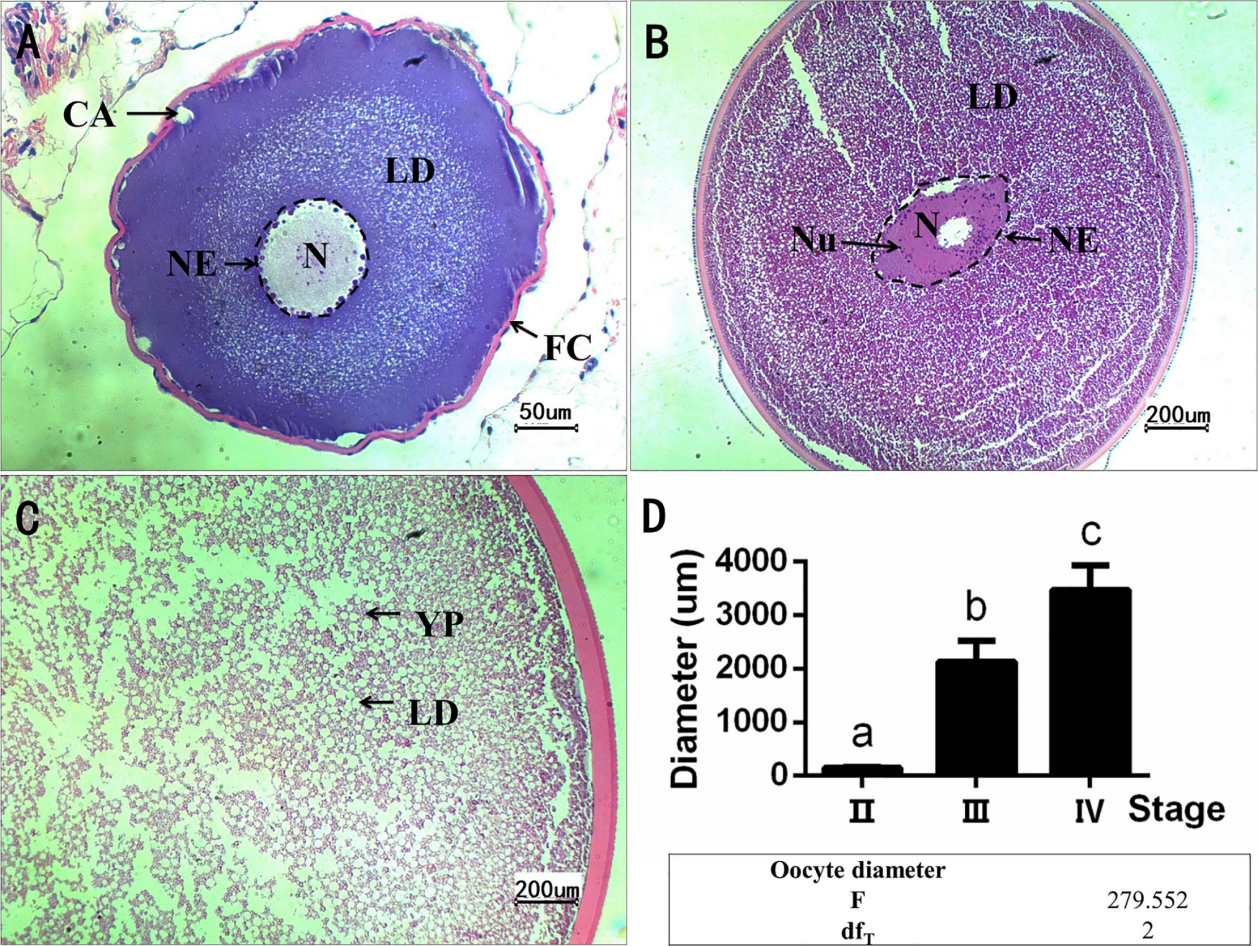
Representative photomicrographs of HE-stained ovary section. A: oocyte at stage II, bars = 50 μm; B-C: part of oocytes at stage III and IV, bars = 200 μm. Abbreviations are cortical alveoli (CA), follicle cell (FC), nuclear (N), nucleolus (Nu), nuclear envelope (NE, indicated by a black dashed box), lipid droplet (LD), yolk protein (YP). D: oocyte diameter at stage II, III, and IV, respectively. Values are expressed as mean ± SEM (n = 4, each group contains 4 fish, and in the gonad of each fish, 3 oocytes were randomly selected to measure the diameter). Different letters indicate significant differences among groups (*P* < 0.05).

Oocytes changed their size and structure according to the developmental stages (Fig 1). At stage II, primary oocytes are 117.9–186.78 μm in diameter, exhibited some cortical alveoli and a layer of follicular cells at the edge and complete nuclear in the center (Fig 1A). At stage III, the oocytes showed bigger diameter (1500–2700 μm) with yolk granules and lipid droplets scattering in the cytoplasm, the nucleus also grew larger and showed the irregular shape (Fig 1B). At stage IV, oocytes further increased the volume to a diameter of 2800–4000 μm, and showed polarized ooplasm filled with light round lipid droplets and dark rice-like yolk proteins (Fig 1C).

### Changes in lipid content of muscle during ovarian development

**Fig 2:**
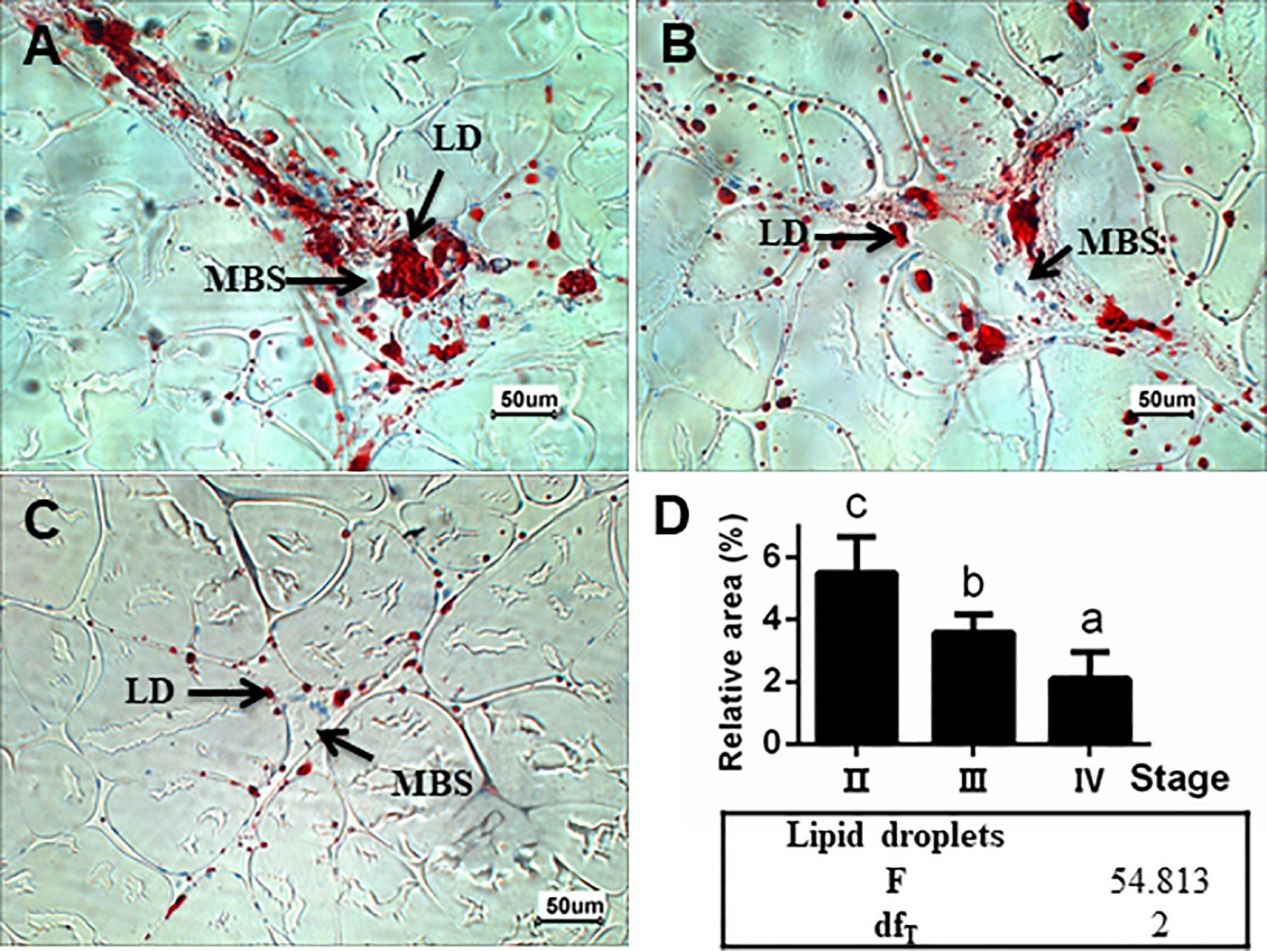
Representative photomicrographs of oil-red O-stained muscle section. A-C: the muscle of female Chinese sturgeon at stage II, III, and IV, respectively. Abbreviations are lipid droplet (LD), muscle blood sinus (MBS). Bars = 50 μm. D: relative area of lipid droplets in oil-red O-stained muscle area of Chinese sturgeons at stage II, III and IV, respectively. Values were expressed as mean ± SEM (n = 4, each group contained 4 fish, and in the muscle slices from each fish, 3 images of 200-fold field of vision were randomly selected to measure the relative area of the lipid droplets). Different letters indicate significant differences among groups (*P* < 0.05).

In muscle of female Chinese sturgeons, the area percentage of lipid droplet decreased significantly (*P* < 0.05) as the ovary development proceeded from stage II to stage IV (Fig 2).

### Changes in serum biochemical components during ovarian development

**Fig 3:**
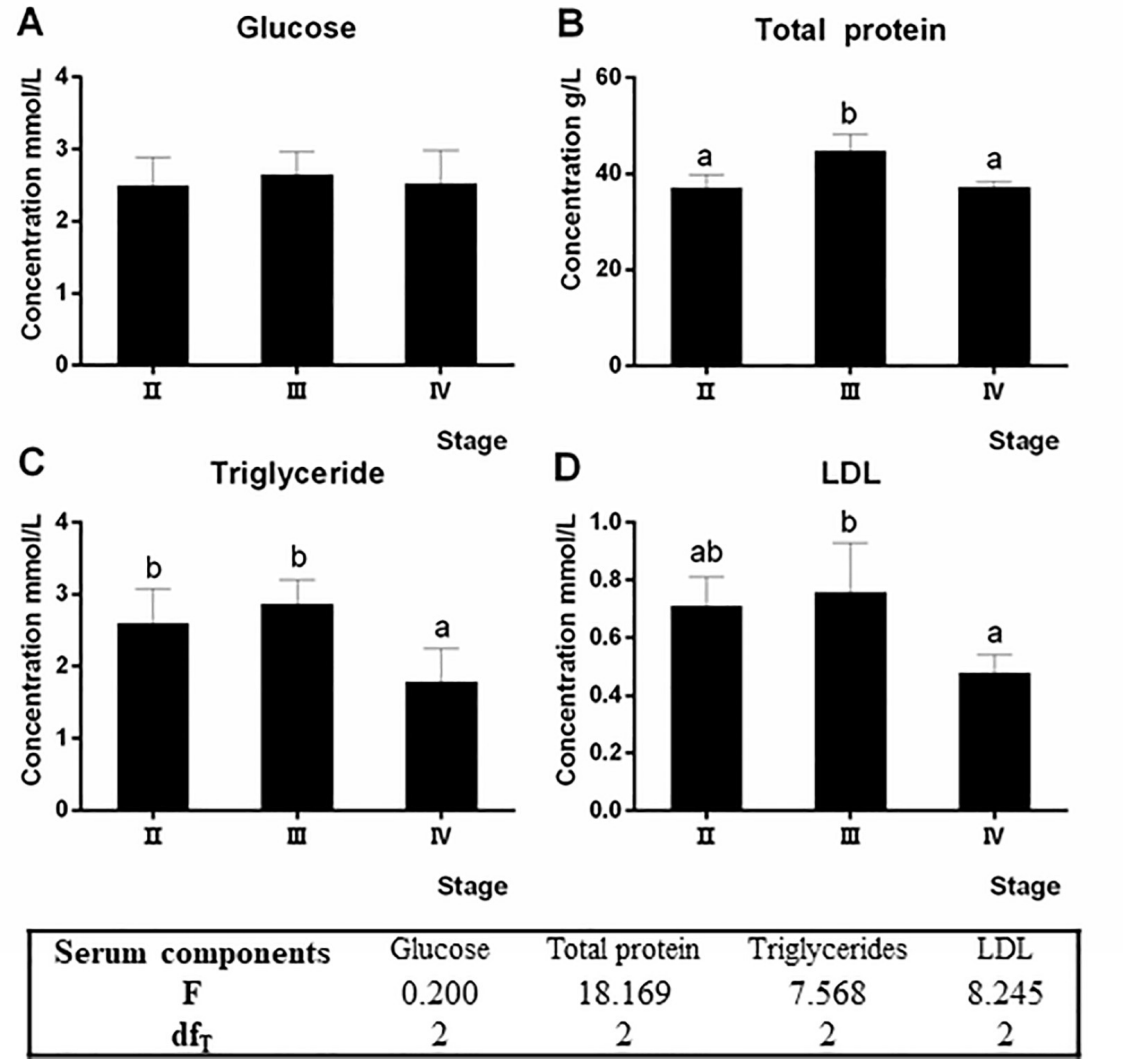
Changes of serum biochemical components of Chinese sturgeons at different developmental stages. Values were expressed as mean ± SEM (n = 4). Different letters indicate significant differences among groups (*P* < 0.05).

The serum glucose content did not vary at different developmental stages (Fig 3A). The serum total protein at stage III was significantly higher than that of stage II and IV (*P* < 0.05) (Fig 3B). The contents of triglycerides and LDL in serum were significantly higher at stage II and stage III than those at stage IV (*P* < 0.05) (Fig 3C and 3D).

### Serum metabolic profiles

#### Raw data analysi

10051 metabolite peaks (6470 peaks in ESI+ mode and 3581 peaks in ESI- mode) were selected from the final data set for subsequent analysis after the acquired raw data was preprocessed. The score plot of PCA showed three clusters correspondent to those three different groups (ovarian stages) of serum in positive and negative ion scan modes, respectively (S1 Fig). The data in each group exhibited difference from the other two groups in the PCA score plot. Besides, the PLS-DA scores plots and permutation tests (S2 Fig) showed clear separation between each two groups, as R^2^ and Q^2^ values of real model were always larger than R^2^ and Q^2^ values from permutation tests.

#### Metabolites screening and identification

Between stage II and stage III, there were 291 differential ions in positive mode and 145 differential ions in negative mode, respectively (S1 Table). Between stage III and stage IV, there were 598 differential ions in positive mode and 305 differential ions in negative mode, respectively (S1 Table). By searching KEGG database associated with primary data (parent ions, identification level 1), 211 differential ions (183 up-regulated, and 28 down-regulated) were identified in serum from stage III in comparison with that from stage II, and 385 differential ions (182 up-regulated, and 203 down-regulated) were identified in serum from stage IV in comparison with stage III (S1 Table). The differential ions were also identified by searching fragmentation available from KEGG database (identification level 2). S1 Table summarized additional details of the differential ions between groups.

**Fig 4:**
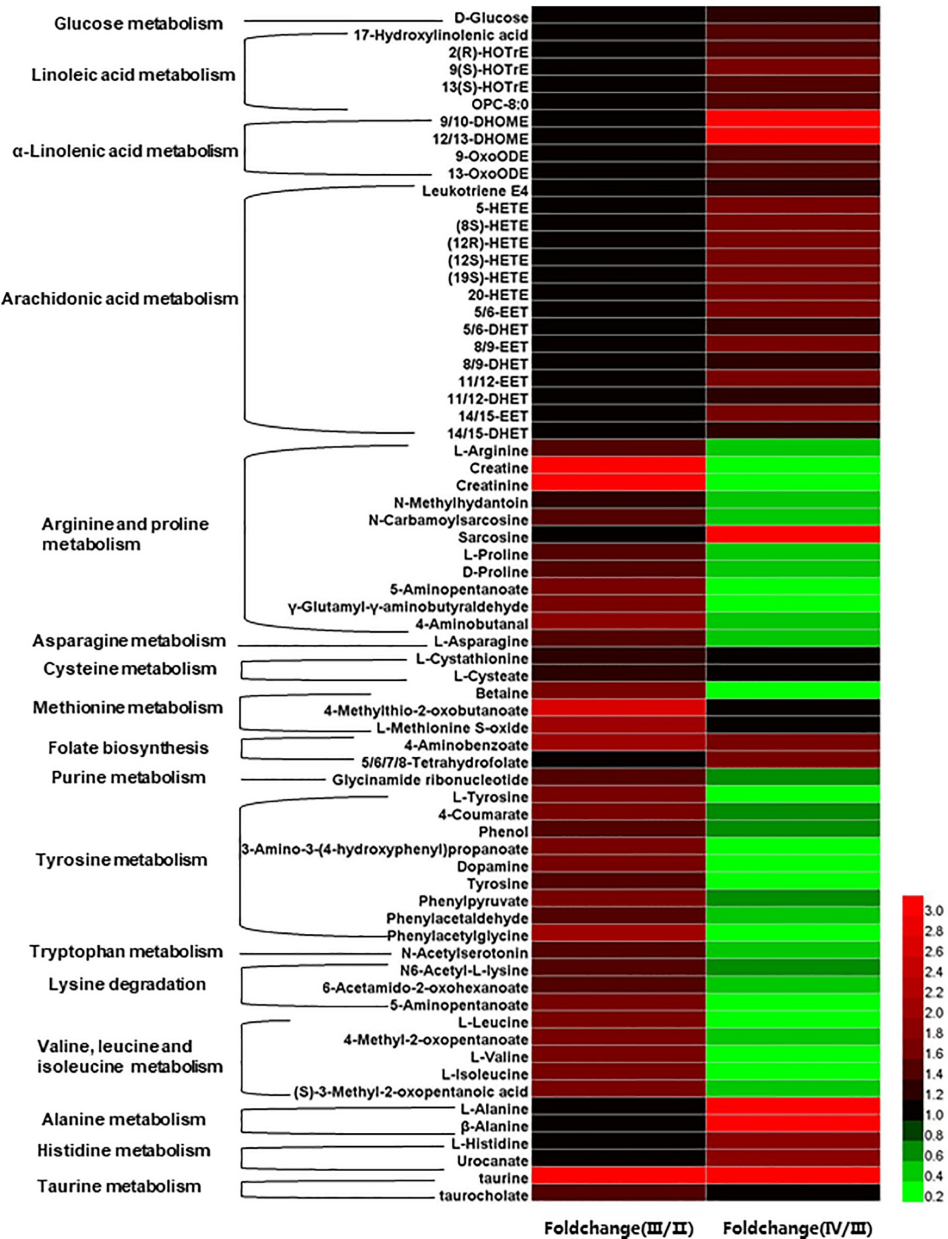
Heat map of differentially expressed metabolites with annotating to KEGG pathway. Values were expressed as fold change relative to the corresponding control.

The heat map of serum differential metabolites annotated to KEGG pathway (Fig 4) showed the most oxylipins derived from linoleic acid metabolism pathway, whilst α-linolenic acid metabolism pathway and arachidonic acid metabolism pathway exhibited no significant difference between stage II and stage III, but they were significantly higher at stage IV than those at stage III (*P* < 0.05). Remarkably, the contents of 9/10-DHOME and 12/13DHOME at stage IV were 6 times higher than at stage III.

Differential metabolites of arginine, cysteine, methionine, purine, tyrosine, lysine degradation pathways and valine, leucine and isoleucine metabolism pathway, were significantly higher at stage III than those at stage II (*P* < 0.05), but significantly lower at stage IV than those at stage III (*P* < 0.05). Sarcosine and metabolites of alanine and histidine were significantly higher at stage IV than those at stage III (*P* < 0.05). Especially, creatine and creatinine at stage III were 3 times higher than those at stage II, and differential metabolites of folate and taurine pathways always increased with ovary development.

### Expressions of genes related to lipid, yolk protein, and amino acid metabolism in ovary

**Fig 5:**
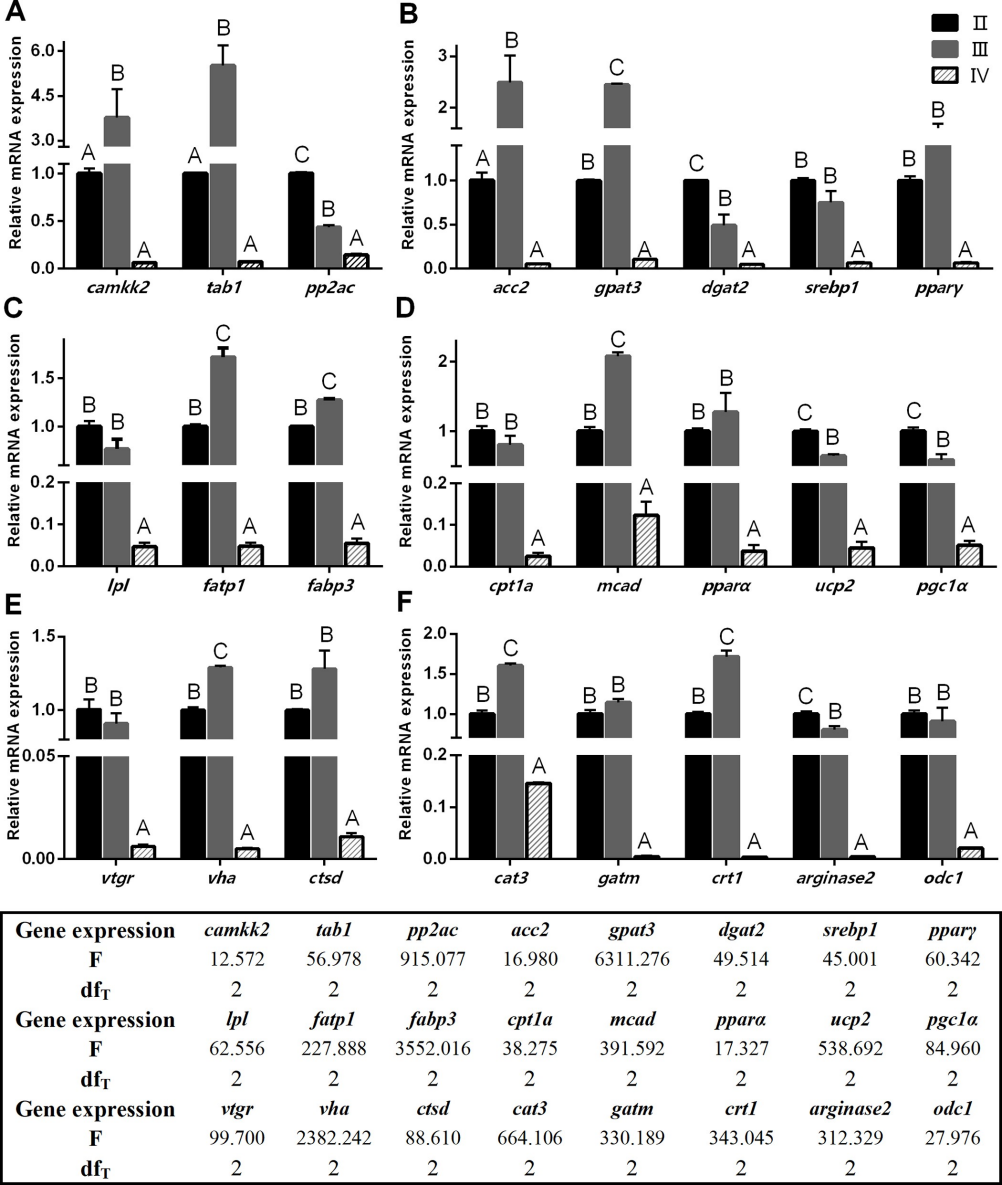
Expressions of gene related to lipid and amino acid metabolism in ovary at different developmental stages. A-D: genes related to the upstream stimulator of AMPK, lipid biosynthesis, lipid transport and absorption, lipolysis and oxidation, respectively. E-F: genes related to yolk protein deposition and arginine metabolism, respectively. Values were means ± SEM (n = 4), and expressed as fold change relative to the corresponding control. Above the same gene bars, different letters ABC indicate significant differences (*P* < 0.05) among various stages.

Regarding to AMPK pathway in ovary, the mRNA expressions of 2 upstream stimulators, calcium/calmodulin-dependent protein kinase kinase 2 (*camkk2*) and TGF-beta activated kinase 1 binding protein 1 (*tab1*) at stage III, were significantly higher than those at stage II and stage IV (*P* < 0.05) (Fig 5A). However, the mRNA expression of inhibitor, serine/threonine-protein phosphatase 2A catalytic subunit (*pp2ac*), was the highest at stage II, and decreased with ovary development (*P* < 0.05).

In lipid biosynthesis pathway, the mRNA expressions of acetyl-coA carboxylase 2 (*acc2*) and mitochondrial glycerol-3-phosphate acyltransferase 3 (*gpat3*) at stage III were significantly higher than those at stage II and stage IV (*P* < 0.05) (Fig 5B). The mRNA expression of diacylglycerol O-acyltransferase 2 (*dgat2*) was the highest at stage II, and significantly decreased with ovary development (*P* < 0.05). The mRNA expressions of transcription factors, sterol regulatory element-binding protein 1 (*srebp1*) and peroxisome proliferator-activated receptor γ (*pparγ*) at stage II and stage III were significantly higher than those at stage IV (*P* < 0.05).

Regarding the transport of lipid into oocyte, the mRNA expression of lipoprotein lipase (*lpl*) at stage II and stage III was significantly higher than that at stage IV (*P* < 0.05) (Fig 5C). The mRNA expressions of fatty acid transport protein 1 (*fatp1*) and fatty acid binding protein 3 (*fabp3*) at stage III were significantly higher than those at stage II and stage IV (*P* < 0.05). With respect to the lipid oxidation, the mRNA expressions of carnitine O-palmitoyltransferase 1A (*cpt1a*) and peroxisome proliferator-activated receptor α (*pparα*) at stage II and stage III were significantly higher than those at stage IV (*P* < 0.05) (Fig 5D). However, the mRNA expressions of medium-chain acyl-coA dehydrogenase (*mcad*) at stage III was significantly higher than those at stage II and stage IV (*P* < 0.05), whilst the mRNA expressions of mitochondrial uncoupling protein 2 (*ucp2*) and pparγ coactivator-1α (*pgc1α*) at stage II were significantly higher than those at stage III and stage IV (*P* < 0.05).

In yolk protein deposition pathway, the mRNA expressions of vitellogenin receptor (*vtgr*), vacuolar-type H^+^-ATPase (*vha)*, and cathepsin d (*ctsd*) at stage II and stage III were significantly higher than those at stage IV (*P* < 0.05) (Fig 5E). In arginine metabolism pathway (Fig 5F), the mRNA expressions of cationic amino acid transport protein 3 (*cat3*) and creatine transporter 1 (*crt1*) at stage III were significantly higher than those at stage II and stage IV (*P* < 0.05). The mRNA expressions of glycine amidinotransferase (*gatm*) and ornithine decarboxylase 1 (*odc1*) at stage II and stage III were significantly higher than at stage IV (*P* < 0.05). The mRNA expression of *arginase 2* at stage II was significantly higher than that at stage III and IV (*P* < 0.05).

## Discussion

This study investigated the physiological and metabolic changes of captive-bred female Chinese sturgeon at different ovarian stages. Similar to other species, the oocytes of Chinese sturgeon greatly changed their size and structure when developing from stage II to stage IV, indicating a myriad of physiological and metabolic processes involved in [22], including the energy and protein allocation to the ovary through lipid and vitellogenin transport [15]. Fasting blood samples could reflect the body physiological and health status in mammals [2–4]. The metabolome in fasting serum from Chinese sturgeon at different ovarian stages supported the metabolic changes of fish body in harmony with ovarian development.

In this study, with the increase of egg size and lipid droplets in the ovary histology, lipid droplets in muscle sections decreased, which suggest the consumption of muscle energy/lipid reservoir during ovarian development from stage II to IV. Our previous study stated that Chinese sturgeon fed with higher lipid level diet could develop their ovaries to stage III or IV while they showed constant body weight and decreasing muscle lipid droplets [24]. These suggest that the energy required for ovary development in this fish is of dual source, including exogenous food and endogenous sources (liver, muscle and visceral fat), as reported in other fish species [6, 23]. Furthermore, energy transfer from somatic tissue to gonad in capital-breeding fish [24] and DHA migration from liver/muscle to ovary in zebrafish (*Danio rerio*) [25] have been verified. Whether direct lipid and DHA transfer from muscle to ovary occur in Chinese sturgeon deserves further investigation.

**Fig 6:**
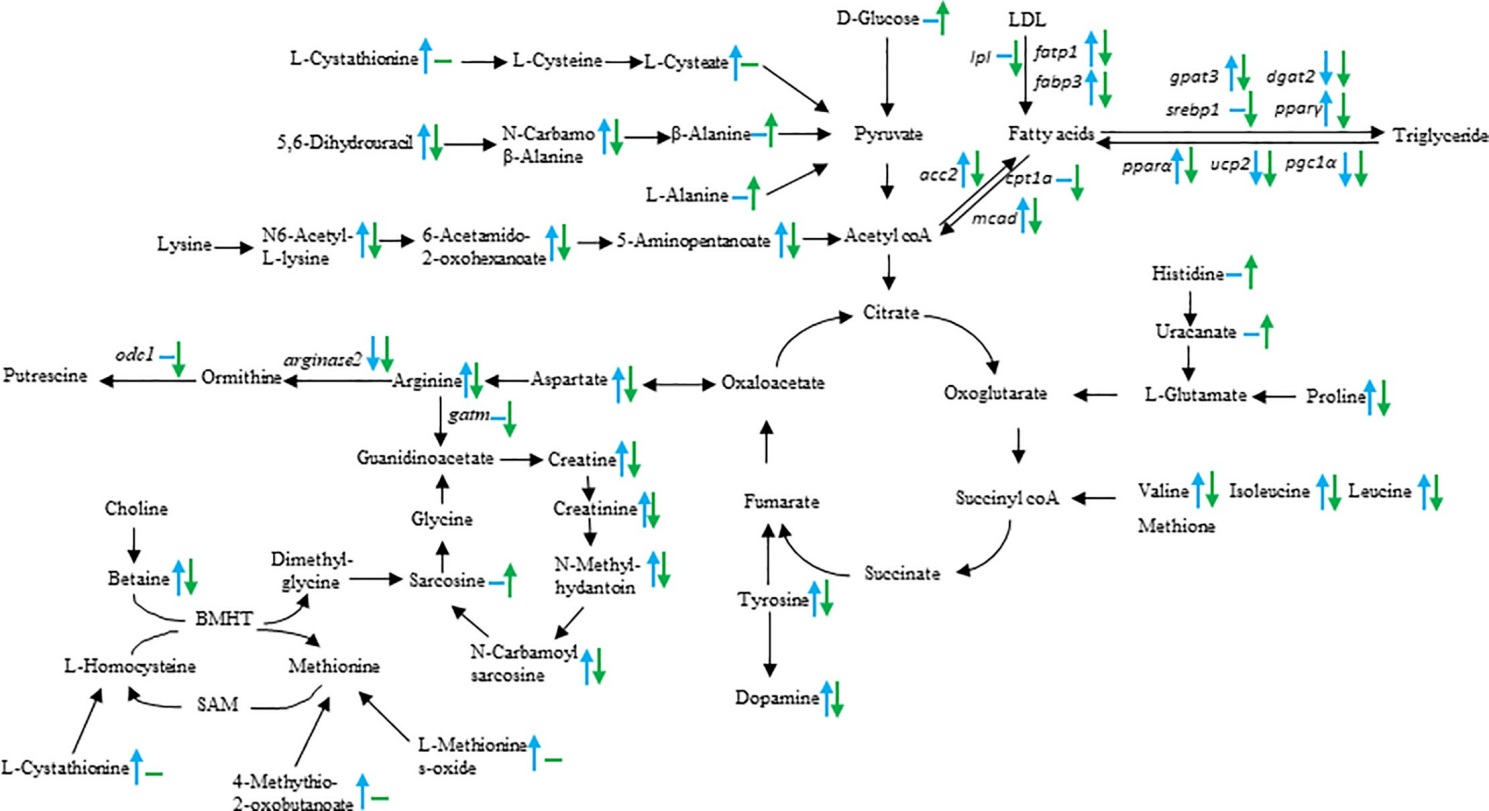
The stages of ovarian development influenced on the metabolic pattern, represented by changes in metabolites and related gene expression levels. Blue arrows: stage III vs stage II; Green arrows: stage IV vs stage III. The upward arrow indicates upregulated; downward arrow indicates downregulated; horizontal line indicates no significant difference. LDL: Low density lipoprotein; BHMT: Betaine-Homocysteine S-Methyltransferase.

When the energy and protein were allocated to the developing ovaries of Chinese sturgeon, ovarian metabolism showed dynamic changes at molecular level. Consistent with the histological observations, the alterations of *lpl*, *fatp1*, *fabp3*, *cpt1a*, and *mcad* (Fig 6) revealed a large amount of exogenous lipid was absorbed into ovaries from stage II to stage III, and then oxidized for energy in oocytes at stage III. The changes in lipid accumulation and consumption responding to the ovary stages were similar to those in mud crabs and pregnant dairy cows [26, 27]. In addition, when combining expression patterns of genes related to lipid synthesis (*acc2*, *gpat3*, *dgat2*, *srebp1*, and *pparγ*) with those related to lipolysis (*pparα*, *ucp2*, *pgc1α*) (Fig 6), this study rationalized that lipogenesis and lipolysis were both in active dynamics at stage II and stage III during lipid accumulation, which was supported by the relatively higher serum lipids data. Vitellogenin, the precursor of yolk proteins, is the main source of amino acids for oocyte maturation and larvae development [28]. The amino acids derived from the yolk protein via cathepsins catalysis may also act as the osmotic driving force of hydration during oocyte maturation [29]. Consistent with the histological changes, ovarian expression patterns of *vtgr*, *vha*, and *ctsd* revealed that a large amount of vitellogenin was absorbed into oocytes from stage II to stage III, and then was mainly hydrolyzed to yolk proteins from stage III to stage IV.

As indicated by serum metabolites, Chinese sturgeon changed body metabolism to meet the demand of ovary development. Total protein, triglycerides, and LDL contents in serum could reflect body nutritional status and metabolic activity [30]. What’s more, serum total protein and triglyceride contents could be modeled to predict the ovarian maturation stages [31]. The increases of serum total protein, triglycerides, and LDL contents under the same culture condition might indicate the strengthened metabolic activity of fish body adapting to the ovarian accumulation of yolk proteins and lipids during ovary development from stage II to stage III. Similar serum biochemical changes responding to ovary development in English sole *Parophrys vetulus* supported our results [31].

Serum metabolomics in this study further indicated Chinese sturgeon broodstocks finely regulated their body metabolism in response to ovarian development. Oxylipins, the products of polyunsaturated fatty acids oxidation, could affect the physiological processes in a broad range, including adipogenesis, cell proliferation and inflammation [32, 33]. In this study, most serum oxylipins (especially 9/10-DHOME and 12/13-DHOME) derived from α-linolenic acid, linoleic acid or arachidonic acid exhibited the highest values in Chinese sturgeon at stage IV, which suggests their potential function at this stage and deserves future study.

The function of amino acids during the ovarian development received little attention. The differential metabolites in various amino acid metabolism pathways identified in this study suggest their possible roles in ovarian development. Arginine acts as the precursor of nitric oxide, polyamine, and creatine synthesis for different physiological functions [34]. Most metabolites in arginine metabolism pathway increased from stage II to stage III, including arginine, proline, γ-aminobutyric acid related metabolites, and creatine related metabolites, which indicated the enhanced arginine metabolism is a biomarker for the ovary at stage III. Especially, arginine is both the product of oxaloacetate (via aspartate) and the precursor of putrescine (via ornithine) and creatine (via guanidinoacetate) (Fig 6). The high serum aspartate content and high ovarian expression of *arginase2* and *odc1* at stage II and III indicated the active synthesis of polyamines, including putrescine and spermidine via the arginine metabolism pathway, for the control of cell proliferation and differentiation during earlier ovarian development [35]. Besides, as the cell energy shuttle (phosphocreatine can regenerate ATP), creatine plays a critical role in regulating energy metabolism [36]. The high serum contents of creatine related metabolites and the high ovarian mRNA expressions of *gatm* (encoding glycine amidinotransferase) and *crt1* (encoding creatine transport protein) at stage II and III indicate the active creatine supply to the ovary, which might be a metabolic response to the high energy demand of developing ovaries.

From stage II to III, serum levels of betaine (the precursor of methionine) and metabolites in folate biosynthesis and purine metabolism pathways all significantly increased, indicating the active one-carbon metabolism in response to the vitellogenic process. Amazingly, the arginine metabolism pathway also linked to the one-carbon metabolism in this study, as sarcosine in arginine metabolism pathway is importantly involved in maintaining methyl balance [37]. Folate plays a fundamental role in regulating DNA and histone methylation [38]. The female Chinese sturgeon showed the continuously increasing levels of folate related metabolites in serum during oocyte maturation and the increased sarcosine level at stage IV, which implied the importance of methylation in active nucleic acid synthesis.

To date, the aromatic amino acids are only known as precursors for the monoamine neurotransmitters, serotonin and catecholamines in brain, besides their roles as protein constituents. Changes in blood concentrations of these amino acids alter the formation and release of these monoamine transmitters [39]. The role of catecholamines in neuroendocrine control of the reproductive axis has also been recognized in fish [40]. In this study, serum contents of tyrosine and its intermediates, dopamine, as well as N-acetylserotonin (the precursor of melatonin, from tryptophan) all changed with ovarian development stages and showed the highest values at stage III, which means their potential functions in neuroendocrine control of Chinese sturgeon reproduction. In addition, the higher contents of serum lysine, branched amino acids and their metabolites of fish at stage III received our interest due to their possible functions in protein synthesis in oocytes [41] and physiological regulation, including mTOR signaling pathway and lipolysis [42] (Fig 6). Meanwhile, the high serum levels of alanine, histidine and taurine at stage IV also deserve attention as alanine and histidine may participate in the production of carnosine, a dipeptide to scavenge oxygen free radical [43], and taurine may function in the osmotic regulation during oocyte hydration [44].

It seems that an energy-sensing pathway may be involved in the dynamics of lipid accumulation and consumption. The adenosine 5’-monophosphate-activated protein kinase (AMPK), an energy monitor, could be activated to stimulate the catabolism of lipid [45]. The significantly higher expression of *pp2ac*, an AMPK inhibitor, in the ovary at stage II indicated the energy deposit in oocytes for later development by suppressing lipid catabolism. After then, the significantly higher expressions of AMPK activators, *camkk2* and *tab1*, in the ovary at stage III indicated sufficient energy was stored in oocytes, and then the AMPK was triggered to activate other pathways for ovary maturation. In addition, higher contents of some amino acids in circulating system at stage III indicated that their preparation for ovary development, and ovarian gene expressions relating to amino acids metabolism confirmed these amino acids might be absorbed into ovary and then enter the energy metabolism system via pyruvic acid, acetyl-coA, or intermediate metabolites of TCA (Fig 6). The high expression level of *cat3* at stage II and III also proved the statement.

Our study has limitations, in particular the tissue sampled for metabolomics and lipid consumption. Although the metabolites in ovary or liver would be a better choice to examine the metabolic status, this is not applicable due to the limitation of ovarian or hepatic sampling. We should try our best to assure the survival of the adult in the study according to the approval from the government. Compared to other tissues, plasma samples possess a great diversity of biochemical compositions, bring the minimal damage to fish, and ensure the subsequent normal survival of fish. Therefore, serum metabolomics analysis was conducted for the preliminary identification of metabolic changes during ovarian development. Future study should focus on metabolites in ovaries or livers for accurate elaboration of metabolic changes when essential samples could be obtained for the analysis.

In conclusion, the results revealed the active amino acids and lipid metabolism in response to the high energy input during ovarian development from stage II to stage III, and preliminarily discussed the potential functions of alanine, histidine, taurine, folate and oxylipins at ovary stage IV. Hopefully, this study would give an insight into reproductive biology in female Chinese sturgeon.

## Supporting information

S1 Table Identification of different ions. ^a^The number of all differential m/z between two different groups, which including identified ions and unable identified ions. ^b^The number of identified ions by searching KEGG database associated with primary data (parent ions). ^c^The number of identified ions by searching fragmentation available from KEGG database.
(DOCX)

S1 Fig The principal component analysis (PCA) score plots of serum samples collected from stage II, III and IV in positive (A) and negative (B) ion scan modes.
(PDF)

S2 Fig The partial least-squares-discriminant analysis (PLS-DA) score plots of serum samples collected from stage II and stage III in positive (A) and negative (B) ion scan modes, as well as collected from stage III and stage IV in positive (C) and negative (D) ion scan modes.
(PDF)
