# Supplementary material for: Metabolomics and gene expressions revealed the metabolic changes of lipid and amino acids and the related energetic mechanism in response to ovary development of Chinese sturgeon (Acipenser sinensis)
Source: PLoS One. 2020 Jun 26;15(6):e0235043. doi: 10.1371/journal.pone.0235043 (PMC7319304; doi:10.1371/journal.pone.0235043)
Supplement: S1 Table — aThe number of all differential m/z between two different groups, which including identified ions and unable identified ions. bThe number of identified ions by searching KEGG database associated with primary data (parent ions). cThe number of identified ions by searching fragmentation available from KEGG database. (DOCX) [file pone.0235043.s001.docx]

| Differential ions | | | | | Metabolites identification | | | | | |
| --- | --- | --- | --- | --- | --- | --- | --- | --- | --- | --- |
| Comparison among groups | Detect mode | Total ion number^a^ | Up-regulated number | Down-regulated number | Identification level 1^b^ ions number | | | Identification level 2^c^ ions number | | |
|  |  |  |  |  | Total | Up-regulated | Down-regulated | Total | Up-regulated | Down-regulated |
| Stage Ⅱ vs. Stage Ⅲ | Positive ion | 291 | 244 | 47 | 167 | 149 | 18 | 95 | 84 | 11 |
|  | Negative ion | 145 | 97 | 48 | 44 | 34 | 10 | 17 | 12 | 5 |
| Stage Ⅲ vs. Stage Ⅳ | Positive ion | 598 | 279 | 319 | 277 | 123 | 154 | 163 | 79 | 84 |
|  | Negative ion | 305 | 166 | 139 | 108 | 59 | 49 | 49 | 31 | 18 |

**Table 1 Identification of different ions**

^a^The number of all differential m/z between two different groups, which including identified ions and unable identified ions.

^b^The number of identified ions by searching KEGG database associated with primary data (parent ions).

^c^The number of identified ions by searching fragmentation available from KEGG database.
