# Supplementary figures and images for: Metabolomics and gene expressions revealed the metabolic changes of lipid and amino acids and the related energetic mechanism in response to ovary development of Chinese sturgeon (Acipenser sinensis)
Source: PLoS One. 2020 Jun 26;15(6):e0235043. doi: 10.1371/journal.pone.0235043 (PMC7319304; doi:10.1371/journal.pone.0235043)

**A**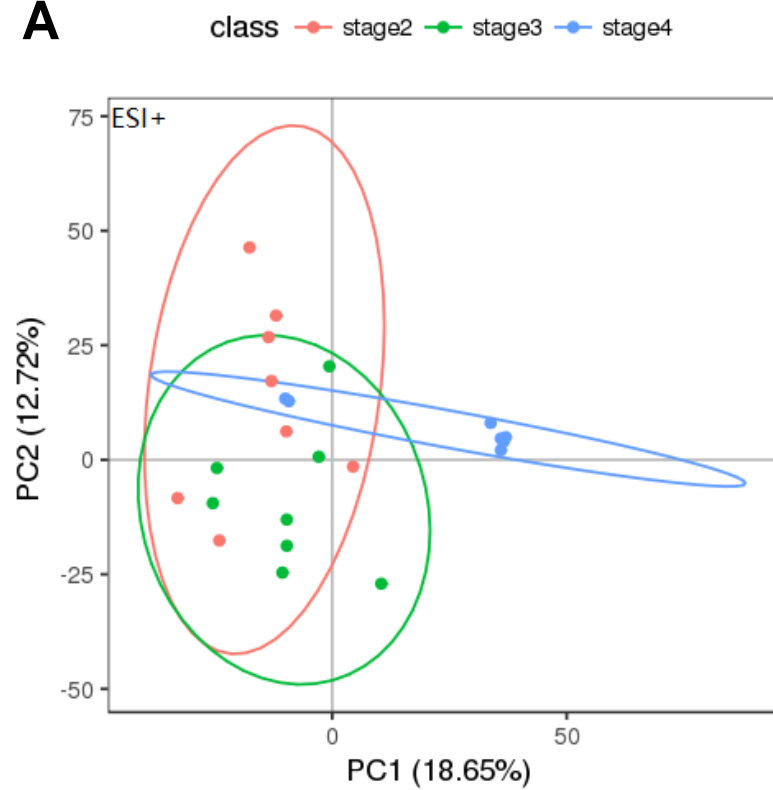**B**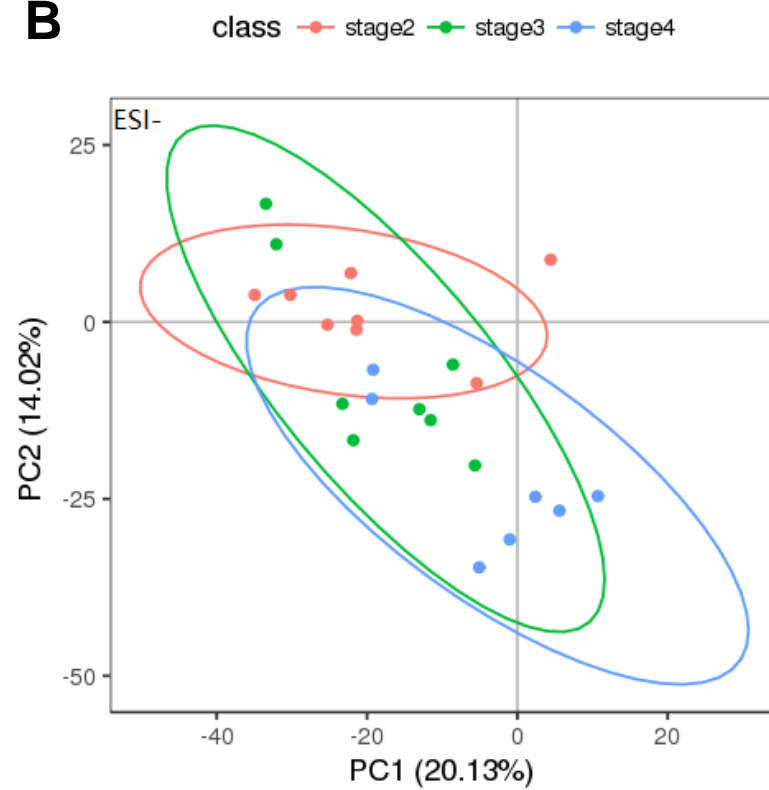

Supplement: S1 Fig — The principal component analysis (PCA) score plots of serum samples collected from stage II, III and IV in positive (A) and negative (B) ion scan modes. (PDF) [file pone.0235043.s002.pdf]

**A** $R^2=0.9209$   $Q^2=0.2847$ 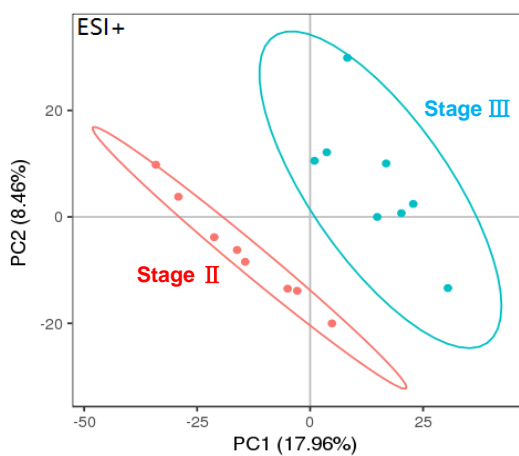**B** $R^2=0.8756$   $Q^2=0.32$ 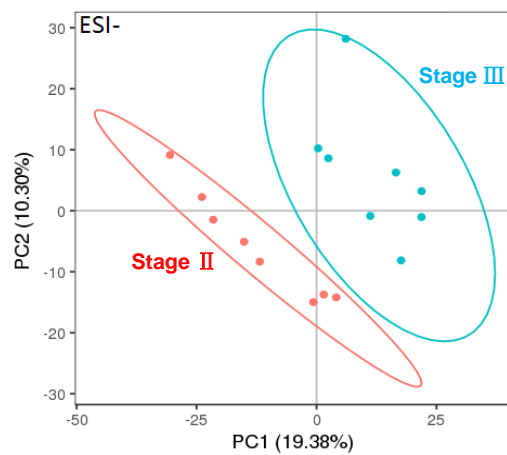**C** $R^2=0.9858$   $Q^2=0.7839$ 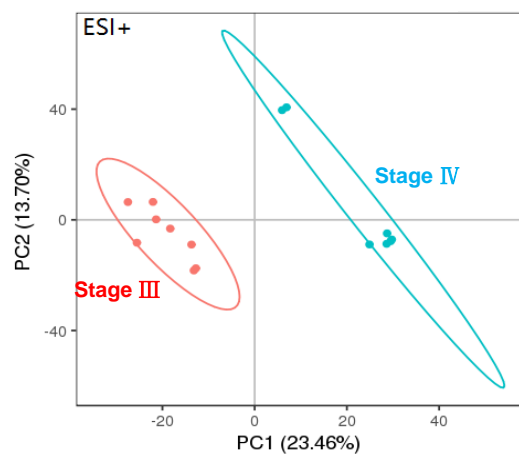**D** $R^2=0.9825$   $Q^2=0.6804$ 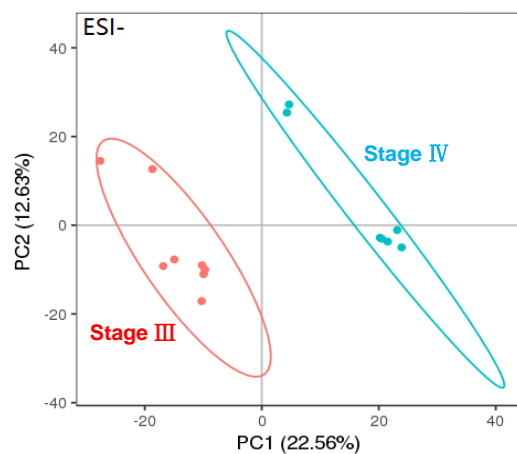

Supplement: S2 Fig — The partial least-squares-discriminant analysis (PLS-DA) score plots of serum samples collected from stage II and stage III in positive (A) and negative (B) ion scan modes, as well as collected from stage III and stage IV in positive (C) and negative (D) ion scan modes. (PDF) [file pone.0235043.s003.pdf]
